# Genomic and Epigenomic Advances in Hearing Loss: Molecular Mechanisms, Diagnostics, and Emerging Therapies

**DOI:** 10.3390/jpm16060306

**Published:** 2026-06-04

**Authors:** Giuseppe Alberti, Francesco Galletti, Daniele Portelli, Cosimo Galletti, Sabrina Loteta, Bruno Galletti, Mario Lentini, Salvatore Ronsivalle, Salvatore Maira, Jerome Rene Lechien, Quentin Mat, Antonino Maniaci

**Affiliations:** 1 Unit of Otorhinolaryngology, Department of Adult and Development Age Human Pathology “Gaetano Barresi”, University of Messina, 98122 Messina, Italy; galberti@unime.it (G.A.); francesco.galletti@unime.it (F.G.); daniele.portelli09@gmail.com (D.P.); lotetas@unime.it (S.L.); bruno.galletti@unime.it (B.G.); mario.lentini@unikore.it (M.L.); salvatore.maira@unikore.it (S.M.); 2 Department of Medicine and Surgery, University of Enna Kore, 94100 Enna, Italy; cosimo.galletti01@unikore.it; 3 Ospedale Gravina Caltagirone Asp 3 Catania, 95123 Catania, Italy; salvatore.ronsivalle@asp.ct.it; 4 Division of Laryngology and Broncho-Esophagology, Department of Otolaryngology-Head Neck Surgery, EpiCURA Hospital, UMONS Research Institute for Health Sciences and Technology, University of Mons (UMons), 7000 Mons, Belgium; jerome.lechien@umons.ac.be (J.R.L.); quentin.mat@umons.ac.be (Q.M.)

**Keywords:** hearing loss, epigenetic mechanisms, DNA methylation, histone modifications, prevention, treatment strategies, hearing health, translational research

## Abstract

**Background:** Hearing loss is a widespread sensory disorder affecting over 1.5 billion people worldwide, with the number projected to exceed 700 million by 2050. It imposes social and economic burdens across all ages and regions. Approximately half of adult cases are preventable, but the underlying causes are complex, with 75–80% due to autosomal recessive genetic factors and key roles for mutations in genes such as GJB2. Advances in sequencing technologies have accelerated gene discovery, but challenges remain in interpreting variants. Epigenetic mechanisms such as DNA methylation and histone modifications are increasingly recognized as crucial in auditory biology and could offer new biomarkers and therapeutic targets. Integrating epidemiological, genetic, and epigenomic data is essential to developing targeted prevention and treatment strategies to reduce the global burden of hearing loss. **Methods:** This narrative review examines recent genomic and epigenomic advances in hearing loss, with particular emphasis on molecular mechanisms, emerging diagnostic applications, and translational therapeutic opportunities. A comprehensive review of current epidemiological data, genetic studies, and epigenomic research was conducted using the peer-reviewed literature from international databases. Key areas of interest include inheritance patterns, molecular pathways, and recent advances in omics technologies. **Results:** Epigenetic mechanisms, including DNA methylation and histone modifications, are increasingly recognized as important regulators of cochlear development and hair cell survival, although much of the current evidence remains preclinical. Studies suggest that peripheral epigenetic signatures may serve as biomarkers for early diagnosis and risk stratification. **Conclusions:** Integrating established screening pathways with epidemiological trends and molecular knowledge offers a promising path toward precision medicine in hearing care. Connecting these domains is essential to developing equitable and effective interventions and addressing persistent global disparities in hearing health. This review highlights the evolving landscape of auditory genetics and epigenetics and outlines future directions for translational research and personalized therapy.

## 1. Introduction

Hearing impairment is one of the most common sensory disorders in humans worldwide, regardless of age and geographic region. The World Health Organization estimates that >1.5 billion people, or nearly 20% of the world population, experience some form of hearing loss, and about 430 million have clinically significant disabling impairment [1]. If the current trend continues, this number will surpass 700 million by 2050 [1]. The Global Burden of Disease study has added to the existing burden evidence base [1], and hearing impairment, now affecting >1.57 billion people, is a major contributor to years lived with disability [2]. In children and adolescents younger than 20 years, more than 97 million have hearing loss with otitis media and congenital conditions leading the etiologies [3]. Concurrently, the burden of deafness also continues to be high. By 2021, almost 9.9 million individuals were estimated to live with deafness, and age-standardized prevalence rates have declined slightly, but significant regional disparities remain [4]. These data demonstrate the increasing prevalence of hearing loss and its substantial impact on social functioning, developmental outcomes, and economic productivity. However, age-standardized metrics may underestimate the true population burden due to demographic ageing, particularly in high-income regions where absolute case numbers continue to rise despite stable or declining standardized rates [5]. Despite this growing burden, approximately 50% of adult hearing loss is preventable, highlighting significant public health opportunities for targeted intervention [6]. However, the etiological landscape remains highly heterogeneous, with substantial variation in genetic architecture across populations and age groups. This heterogeneity complicates efforts to develop universal screening and intervention protocols. Autosomal recessive inheritance accounts for 75–80% of genetic hearing loss, while autosomal dominant, X-linked or mitochondrial heritances are less common [6,7].

High-resolution sequencing strategies (whole-exome, whole-genome, and long-read) have accelerated the pace of discovery of rare monogenic as well as common polygenic contributors, though their penetrance, expressivity, and phenotypic correlations vary across diverse populations [8] (Table 1).

Despite advances in gene discovery, several critical challenges persist: (1) variant interpretation remains limited by incomplete population databases and ethnic representation bias, with VUS rates of 30–45% in WES studies; (2) genotype–phenotype correlations show considerable variability even within single-gene disorders; and (3) oligogenic and modifier gene effects are incompletely characterized in most hearing loss cohorts [9,10].

Beyond genomic variants, epigenetic regulation has emerged as an important modulator of auditory biology. Epigenetic mechanisms—including DNA methylation, histone modifications, chromatin remodelling, and non-coding RNA regulation—represent critical layers of auditory gene control. These processes influence cochlear development, hair cell fate determination, stress-response pathways, and regenerative potential.

Critically, epigenetic marks are highly tissue- and time-specific, raising important questions about the validity of peripheral blood signatures as proxies for inner ear biology—a limitation that must be addressed in biomarker development [11,12,13]. However, the epigenetic landscape has been relatively under-explored in auditory science, primarily because of the tissue specificity and changes over time [14]. However, DNA methylation patterns in peripheral blood might reflect the inner ear’s epigenetic changes and provide potential biomarkers for nonsyndromic hearing loss [15], whereas wider systematic reviews exhibit their physiological regulatory functions on hearing impairment and associated pathophysiology, especially in animal models of HL [16]. These initial findings highlight the potential of epigenomic approaches in uncovering novel pathomechanisms.

The development of high-throughput epigenomic technologies has enabled increasingly detailed profiling of epigenetic marks, including CpG methylation and histone acetylation, across specific cell types [17].

Applied to auditory research, these methods hold promise for understanding how environmental factors as ageing, noise exposure, and ototoxic drugs interact with the epigenome to drive gene expression and trajectories of hearing. For instance, animal studies demonstrate how epigenetic modifications support hair cell survival and regeneration, essential mechanisms for combating hearing loss [11].

**Figure 1:**
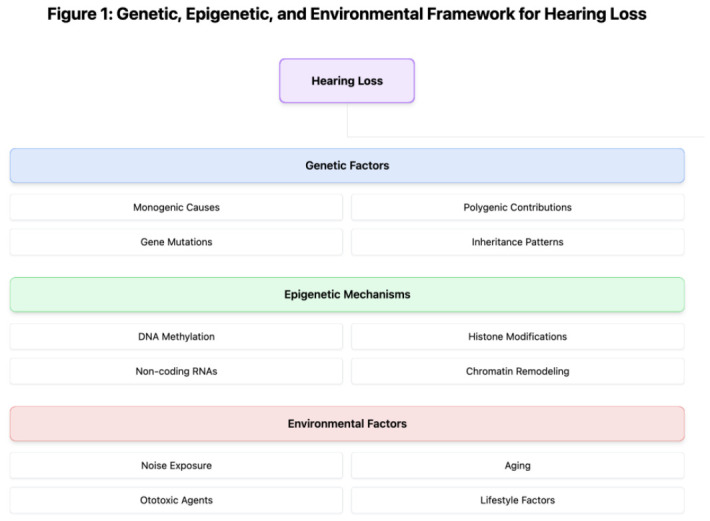
Integrated framework linking global burden, genetic susceptibility, epigenetic regulation, environmental exposure, and precision-medicine opportunities in hearing loss.

Integrating epidemiologic data with genomic and epigenetic insights provides a comprehensive framework for understanding the biological and environmental determinants of hearing loss. The convergence of population-based trends with molecular mechanisms is opening new opportunities for precision prevention, risk stratification, and targeted therapeutic strategies for hearing loss (Figure 1). Against this background, the present review examines how advances in genomics and epigenomics are reshaping current understanding of hearing loss, from molecular pathogenesis to diagnostic innovation and emerging therapeutic approaches. Although epidemiological evidence is considered an important contextual framework, the primary focus is the critical synthesis of genetic and epigenetic mechanisms relevant to precision medicine.

## 2. Materials and Methods

### 2.1. Literature Search Strategy

We performed a structured search across PubMed, MEDLINE, Embase and Google Scholar to identify publications focusing on the genomics and epigenetics of hearing loss. Search criteria comprised “hearing loss and genomics”, “epigenetics”, “DNA methylation”, “microRNA”, and “gene therapy,” as well as multi-omics techniques and cochlear mechanisms. To reflect recency and relevance, the search was restricted to studies from the past 15 years, and preference was given to studies conducted over the last decade.

While this review follows a narrative rather than a systematic approach, we adhered to SANRA (Scale for the Assessment of Narrative Review Articles) guidelines to ensure methodological rigour and transparency in reporting.

The literature search was last updated on 1 May 2026. To improve transparency, a retrospective selection summary was reconstructed from the authors’ search library and reference-management records. The PubMed search was performed using the following core strategy, (“hearing loss” OR deafness OR “sensorineural hearing loss”) AND (genomic* OR genetic* OR epigenetic* OR “DNA methylation” OR “histone modification” OR chromatin OR microRNA OR miRNA OR “gene therapy” OR “multi-omics” OR cochlear), limited to publications from the previous 15 years and prioritizing peer-reviewed articles with direct relevance to genomic, epigenomic, diagnostic, or translational aspects of hearing loss. The search was complemented by targeted searches in MEDLINE, Embase, and Google Scholar, together with manual screening of the reference lists of key reviews and primary studies.

### 2.2. Inclusion and Exclusion Criteria

We covered peer-reviewed original research articles, systematic reviews, clinical studies and high-profile conference papers providing significant molecular understanding or translational application. Excluded from consideration were editorials, commentaries, and non-peer-reviewed sources. Disagreements during initial screening were resolved by two independent reviewers.

Two independent reviewers (GA, DP) screened titles and abstracts, with disagreements resolved through discussion with a third reviewer (AM). We prioritized studies with: (1) well-defined phenotyping criteria, (2) validated molecular methods, (3) diverse population samples, and (4) transparent reporting of effect sizes and confidence intervals. Studies were excluded if they lacked peer review, provided insufficient methodological detail, or presented only preliminary conference abstracts without subsequent full publication.

### 2.3. Review Structure

Acknowledging that this is a narrative (rather than systematic) review, we adhered to SANRA guidelines. Data were extracted for study aim, population or model system, method used (e.g., sequencing platform/epigenetic assays), main findings and translational research context for every included study. The characteristics included effect size, molecular target and treatment regimen for empirical studies. Data were descriptively synthesized, focusing on patterns that emerged and evidence that was encountered repeatedly.

During literature screening, one duplicate citation was removed, and materials sourced from non-scientific publishers included in the reference list were excluded. Ultimately, 84 unique scientific studies were retained, covering multiple relevant research areas including epidemiology, genetic cohort research, and sequencing-based diagnostics. As this work is a narrative rather than systematic review, no formal meta-analysis or risk of bias assessment was conducted. Meanwhile, the screening process was retrospectively documented to comply with SANRA principles, in order to improve the study’s transparency and reproducibility.

## 3. Global Burden of Hearing Loss

Although the present review is primarily focused on molecular and translational advances, a concise epidemiological framework is useful for contextualizing the clinical relevance of hearing loss worldwide.

### 3.1. Prevalence, Geographic Distribution, and Temporal Trends

The burden of hearing loss remains a pressing public health challenge across global communities, amplified by demographic ageing and widening health disparities. Recent data from a Global Burden of Disease analysis underscore that, by 2021, the global prevalence of complete hearing loss had reached approximately 9.9 million cases, even as the age-standardized prevalence rate (ASPR) declined modestly from 134.35 to 117.79 per 100,000 population between 1992 and 2021—indicating marginal gains in reducing standardized rates that mask underlying population growth and ageing dynamics [5].

Geographic patterns reveal stark regional inequities, though interpretation requires caution: ASPR may mask true population burden when demographic structures differ substantially across regions. For instance, while ASPR declined in Sub-Saharan Africa (EAPC −0.74), absolute case numbers likely increased due to population growth—a discrepancy with important implications for resource allocation and service planning [5]. Moreover, East Asia encountered a dramatic reversal, with prevalence rising by over 60%, particularly in high-income Asia–Pacific regions, where ASPR surged nearly 84% [5]. In children and adolescents, estimates indicate more than 97 million individuals under age 20 were affected globally in 2021, translating to around 3.9 million years lived with disability (YLDs), with prevalence increasing gradually (EAPC of +0.15% since 1990) and disproportionately impacting low- and low–middle-SDI regions [18]. Such findings reflect the complex interplay between population dynamics and epidemiological trends among youth populations. In the United States, modelled estimates attribute hearing loss to 72.9 million individuals in 2019—about 22% of the population—consistent with increasing YLD burdens and lower rates of hearing aid utilization, especially among mild cases [19]. Together, these data portray a burden both expansive and intricately stratified, revealing the inadequacy of age-standardized metrics in capturing the growing, real-world toll of hearing impairment.

Temporal trends also warrant critical examination. The reported ASPR decline from 134.35 to 117.79 per 100,000 between 1992 and 2021 represents only a 12% reduction over 29 years—modest progress given substantial investments in newborn screening, hearing aid technology, and public health initiatives during this period [5]. This sluggish improvement suggests that current prevention and intervention strategies may be insufficient to address the full spectrum of modifiable risk factors.

### 3.2. Socioeconomic, Environmental, Occupational, and Developmental, and Quality-of-Life Implications

Hearing loss poses socioeconomic and developmental burdens with broad implications for quality of life throughout the life span. Approximately 80% of individuals with disabling hearing loss live in low- and middle-income countries (LMICs), so barriers to hearing care exacerbate existing disparities related to access to early detection and intervention [1]. Untreated hearing impairment in children compromises speech and language development, educational achievement, and social-emotional functioning, which may go on to compromise these aspects of functioning even into adulthood, with potential for decreased employment opportunities and mental health [18]. In the United States, hearing loss ranks among the most prevalent chronic conditions and contributes to social isolation, reduced cognitive ability, and increased health care costs, which are estimated in the billions annually, yet hearing aid utilization remains below 10% for individuals with mild-to-profound hearing loss [19,20]. Occupational and environmental factors add to the burden. Occupational and recreational noise remain major contributors to preventable hearing loss worldwide, yet regulatory frameworks show marked international variation. While some high-income countries enforce strict occupational exposure limits (e.g., 85 dBA time-weighted average), implementation in low- and middle-income countries remains inconsistent. Furthermore, personal listening device exposure—estimated to affect over 1 billion young adults—remains largely unregulated despite evidence of dose-dependent risk [21]. Mineworkers, construction workers and factory employees have increased reported prevalence, with hearing damage rates as high as 17% in some industries, and evidence of audiometric impairment in a third of those exposed to loud noise [22,23]. Prospective longitudinal research shows that some workers with over a decade of previous noise exposure are 39% more likely to develop incident HL after a decade than unexposed individuals [24]. Also, personal listening habits contribute to early-life risk; based on the rates of reported usage, it is estimated that more than a billion adolescents and young adults worldwide could be at risk of hearing loss due to unsafe listening practices through their personal audio devices (e.g., smartphones) and exposure to damaging levels of sounds in nightclubs and other loud recreational venues potentially causing hearing damage across their lifetime [25]. Additionally, combined exposure to ototoxicants (such as solvents (toluene, styrene), heavy metals (lead, mercury), and asphyxiants (carbon monoxide) can enhance NIHL, particularly in manufacturing contexts where simultaneous exposures act at the level of the cochlea to increase injury [26]. Whether this is because rural populations, and men in particular, have higher occupational or recreational exposure, inducing increased risk of hearing impairment, these findings also suggest that social, environmental and economic context play an important role in the prevalence and severity of hearing loss [27]. Hearing loss affects far more than auditory perception, contributing to developmental delays, reduced productivity, and increased psychological burden, all grounded upon an intricate intermediate network of risk factors that are modifiable and non-modifiable with age-, region-, and socioeconomic status-specific characteristics.

## 4. Genetic Architecture of Hearing Loss

Genomic variation defines inherited susceptibility and disease architecture, whereas epigenetic regulation provides a dynamic layer through which developmental programmes, environmental exposures, and ageing may influence auditory phenotypes. Considered together, these mechanisms offer a more complete framework for understanding both congenital and acquired forms of hearing loss.

### 4.1. Monogenic Causes (Syndromic vs. Nonsyndromic)

Genetic hearing loss, especially in prelingual cases, is primarily monogenic: single-gene defects may account for up to 80% of early-onset deafness [28,29]. Of these, the predominant category is nonsyndromic hearing loss, which encompasses about 70% of prelingual deafness cases, with autosomal recessive inheritance as the most common mode (~80%), followed by autosomal dominant (19%), X-linked (<1%) and mitochondrial (<1%) [29]. GJB2 has long been recognized as the most prevalent NSHL gene worldwide, but recent genomic advances have expanded the catalogue to more than 100 associated genes—including STRC, MYO15A, POU3F4, TMPRSS3, and WFS1—that extend our understanding of genetic deafness [9,30,31,32].

However, GJB2 diagnostic yield varies widely across populations, reaching 50% in some Mediterranean and East Asian cohorts, but <10% in Sub-Saharan African populations. This discrepancy is due to the founder effect, consanguinity rates and ascertainment bias in research series. As a result, gene panel designs are optimized for European ancestry and may not have broad utility in other settings.

Among present sequencing cohorts, the most consistently contributing genes are GJB2, STRC (usually due to biallelic deletions/duplications), OTOF and SLC26A4. The combined diagnostic rate for these four genes is 40–60% in prelingual, severe–profound nonsyndromic SNHL. Specifically, variant types with the highest yield are biallelic loss-of-function SNVs in GJB2 and OTOF, large CNVs in STRC and missense variants affecting pendrin function in SLC26A4. Founder mutations also represent important contributors in population-specific series, additionally contributing to the diagnostic yield.

Jervell and Lange–Nielsen syndrome illustrates pleiotropy: pathogenic variants in KCNQ1 or KCNE1 may be associated with hearing loss in the context of broader multisystem disease, underscoring the clinical value of early genetic diagnosis [33].

Furthermore, several genes that were previously known to be only syndromic have now become nonsyndromic genetic mimics: a recent literature review identified almost 80 such loci reflecting the extensive phenotypic variability and diagnostic complexity in the clinic [11,34,35].

### 4.2. Polygenic Contributions and Complex Inheritance Patterns

Recent sequencing efforts have uncovered polygenic contributions to hearing loss.

Although monogenic causes are predominant in early-onset hearing loss, polygenic inheritance is increasingly identified in age-related and adult-onset pathologies. These have led to dozens of risk loci being identified (48 in one large study) and have focused attention on the stria vascularis and other structures within the cochlear nerve as being crucial for acquired hearing loss [5]. Polygenic risk scores (PRSs) based on these loci are predictive of the trait in ancestrally matched populations, with the top quintile exhibiting 2–3 dB threshold elevations even at a young age. Nevertheless, transferability of PRSs across ancestries has been poor so far: the prediction performance is typically 40–80% lower when using a European-derived score on African or East Asian populations [36,37]. This constraint is due to both genetic architecture contrasts and training data skew, emphasizing the importance of multi-ancestry GWAS projects [6,9,14]. A recent study using the Veterans Affairs cohort (n ≈ 85 K cases and 140 K controls) reported different genetic architecture between sexes in hearing traits, which underscores the significance of population stratification by sex in genetic risk prediction modelling [36].

### 4.3. Genomic Testing Strategies and Clinical Impact in Hearing Loss

#### 4.3.1. Diagnostic Workflow: From Gene Panels to Whole-Genome Sequencing

In current clinical practice, targeted hearing loss gene panels remain the preferred first-line diagnostic test for individuals with well-defined nonsyndromic sensorineural hearing loss (SNHL), particularly in pediatric or congenital cases, owing to their high coverage of established deafness genes and efficient detection of recurrent pathogenic variants [31,38]. Whole-exome sequencing (WES) is generally indicated when syndromic features, progressive disease, or unclear inheritance patterns suggest greater genetic heterogeneity, whereas whole-genome sequencing (WGS) may be considered in unresolved cases, especially when copy-number variants, structural rearrangements, deep intronic variants, mitochondrial variants, or pseudogene-associated regions are suspected [31,39].

Recent evidence also supports the added value of WGS for the detection of STRC duplications, OTOA/OTOAP1 pseudogene-associated variants, and deep intronic splice-altering mutations that may be missed by conventional approaches. Overall, this stepwise diagnostic strategy maximizes diagnostic yield while maintaining cost-effectiveness and limiting unnecessary testing.

#### 4.3.2. Molecular Diagnosis and Clinical Impact

Genomic diagnosis can directly influence patient management and clinical decision-making. Identification of OTOF-related auditory neuropathy may support early referral for cochlear implantation, potentially improving speech and language outcomes. Detection of SLC26A4 variants may prompt vestibular and endocrine evaluation in the context of Pendred syndrome or enlarged vestibular aqueducts. In addition, identification of pathogenic MT-RNR1 variants has immediate therapeutic implications, as aminoglycoside exposure should be avoided [30,31,40].

Across clinical cohorts, genomic testing has been shown to influence prognosis, surveillance strategies, genetic counselling, and treatment pathways in a substantial proportion of patients, although the magnitude of clinical impact varies according to phenotype, ancestry, and testing strategy [31,38].

#### 4.3.3. Variant Interpretation and Current Limitations

Despite major technological advances, variant interpretation remains a significant challenge in clinical genomics. Recent diagnostic cohorts report substantial rates of variants of uncertain significance (VUS), reflecting incomplete population databases, limited functional validation, and ancestry-related underrepresentation [10,31,38].

Standardized frameworks, including ACMG/AMP classification criteria and ClinGen Hearing Loss Expert Panel specifications, have improved interpretive consistency; however, uncertainty persists in cases lacking robust segregation data or functional evidence [40]. These limitations highlight the need for continued refinement of reference databases, functional annotation tools, and ancestry-diverse genomic datasets to support more accurate clinical interpretation. Recent studies also support the additional diagnostic value of WGS for the identification of STRC duplications, OTOA/OTOAP1 pseudogene-associated variants, and deep intronic splice-altering mutations that may be missed by conventional approaches. Overall, a stepwise strategy based on targeted gene panels, followed by WES and subsequently WGS in unresolved cases, appears to maximize diagnostic yield while limiting unnecessary testing.

Genomic variation and epigenetic regulation each have clear functional boundaries, and an integrated research perspective that combines the two can build a comprehensive framework covering both congenital and acquired hearing loss; core pathological factors can converge on the same cochlear pathway, which provides evidence supporting the suitability of this perspective.

## 5. Technological Advances Enabling Genomic and Epigenomic Discovery in Hearing Loss

Technological advances have transformed the molecular investigation of hearing loss by increasing the resolution with which coding, structural, mitochondrial, and regulatory variation can be detected. Targeted next-generation sequencing panels remain efficient for known deafness genes, whereas whole-exome sequencing expands variant discovery across coding regions. However, whole-genome sequencing extends beyond exonic regions covered by WES, providing improved detection of copy-number variants, structural rearrangements, mitochondrial DNA changes, and non-coding regulatory variants [2,30]. Long-read sequencing provides additional advantages for repetitive regions, pseudogene-rich loci, and complex structural variants that are difficult to resolve using short-read approaches.

In prelingual nonsyndromic hearing loss, targeted gene panels achieve diagnostic yields of approximately 30–55%, with whole-exome sequencing contributing an additional 10–15% in panel-negative cases, and whole-genome sequencing providing a further incremental yield of 5–10%, particularly through improved detection of structural variants, mitochondrial alterations, and pseudogene-related disruptions. These differences highlight the importance of selecting sequencing strategies based on clinical context. Accordingly, whole-genome sequencing is particularly useful in auditory neuropathy, enlarged vestibular aqueducts, and cases with suspected structural genomic abnormalities.

Despite differences in diagnostic performance, targeted panels and whole-exome sequencing remain widely used due to their cost-effectiveness and established clinical utility. However, whole-genome sequencing increasingly demonstrates superior sensitivity for structural and non-coding variation, as illustrated by the identification of pathogenic PTPRQ variants missed by exome sequencing [39]. Long-read sequencing further enhances the detection of complex structural variation and repetitive regions, offering improved diagnostic resolution in genetically heterogeneous conditions.

Overall, these high-throughput genomic technologies should be viewed as complementary tools within an integrated diagnostic framework rather than isolated alternatives, collectively reshaping the diagnostic paradigm in hearing loss.

### Genotype–Phenotype Correlations and Variant Interpretation Challenges

It remains a great challenge to establish strong genotype–phenotype correlations. For instance, biallelic non-truncating vs. truncating variants were shown to lead to similar degrees of profound symmetric hearing loss and increased overall risk in more than 400 individuals with MYO15A mutations, underscoring the spectrum of variant-specific effects [19]. A second multicentric study of otoferlin (OTOF)-related hearing loss dissected complex phenotypic profiles in a heterogeneous population, highlighting the requirement for fine phenotype description and genotype-driven prognostication [34].

Several genotype–phenotype correlations that impact prognosis and management have been identified. For instance, OTOF gene variants are highly predictive of auditory neuropathy and very favourable results with early cochlear implantation. TECTA mutations generally are associated with mid-frequency hearing loss, while SLC26A4 mutations can be indicative of EVA-associated fluctuation and progression. These correlations improve prognostic accuracy and guide personalized surveillance strategies.

For other genes, the interpretation of variation is limited by small sample sizes and rare variation. Interrogation of variant interpretations is a significant clinical problem, with VUS rates generally varying from 30 to 45% in WES studies. Use of ACMG/AMP guidelines, complemented by those from the ClinGen Hearing Loss Expert Panel, is critical for their uniform classification. However, incomplete population databases, restricted functional assays and genetic locus specificity hinder interpretation and often necessitate interdisciplinary case analysis. TMPRSS3 is encountered with relatively low patient numbers and extreme phenotype heterogeneity [16]. Moreover, a large systematic meta-analysis of COCH variants (DFNA9) has revealed complex auditory phenotype variability and progression, further emphasizing the significance of cumulative audiometric data in variant interpretation [25]. Across the field, however, the interpretation of variants is also complicated by locus and allelic heterogeneity, whereby different genetic loci present similar phenotypes or multiple mutations in each gene can lead to variable clinical severity—both requiring extensive integrative databases and thoughtful clinical assessment [10].

Demographic and methodological differences between landmark sequencing cohorts are crucial. GJB2 and SLC26A4 are reported more frequently in large East Asian series, whereas MYO15A and TMC1 are found at a higher rate in Middle Eastern consanguineous cohorts. European and North American cohorts show major contributions of STRC CNVs that are detectable predominantly with WGS or array-based CNV tools. Cohort characteristics and types of sequencing can affect diagnostic yield, with a range from 35 to 60 differences underscoring how ancestry, population structure, and analytical techniques contribute to the discovery of genes and diagnostic accuracy.

Long-read sequencing approaches, including PacBio HiFi and Oxford Nanopore platforms, are increasingly being explored for difficult-to-resolve genomic regions, structural variants, and complex haplotypes that may be missed by short-read sequencing in hereditary hearing loss [8]. However, their current clinical role remains investigational, and broader implementation will require further validation, standardization of analytical pipelines, and clearer evidence of incremental diagnostic yield.

## 6. Epigenetic Mechanisms in Hearing Function and Pathology

### 6.1. Roles of DNA Methylation, Histone Modifications, and Chromatin Remodelling

Epigenetic regulation—including DNA methylation, histone modifications, and chromatin remodelling—has emerged as a key layer of auditory gene regulation [12]. DNA methylation mediated by DNA methyltransferases (DNMTs) normally suppresses gene transcription directly through inhibition of binding between transcription factors or the recruitment of methyl-binding domain proteins. The perspective of this mechanism in the auditory system exists in two different ways: it promotes hair cell differentiation during development and later limits mammalian regenerative capacity—an important difference with the potential for impact on regenerative medicine [12,40]. Strikingly, murine studies have demonstrated that adult supporting cells impose DNA methylation to suppress the hair cell gene machinery, thus constraining the regenerative capacity of the mammalian cochlea [13,40] (Table 2).

Systematic reviews have highlighted the regulatory role of both CpG and non-CpG methylation patterns in hearing loss and age-related auditory decline [12,38].

Chromatin dynamics also regulate auditory cell identity; ATP-dependent chromatin remodellers together with histone-modifying enzymes have been implicated in cochlear development and are known to affect reprogramming strategies for hair cell regeneration [12]. In addition, chromatin accessibility determines whether developmental and stress-response genes remain transcriptionally permissive or silenced, while three-dimensional genome architecture influences enhancer–promoter communication across auditory gene networks. Although these mechanisms remain less extensively characterized than DNA methylation in the inner ear, emerging data suggest that they are essential for understanding cell-type-specific transcriptional programmes and may become relevant targets for regenerative strategies.

Together, these mechanisms are integrated into an organizing epigenetic scaffold of cochlear development and dysfunction [41,42,43].

### 6.2. Non-Coding RNAs (miRNAs, lncRNAs, etc.) in Auditory Development and Maintenance

Non-coding RNAs, such as miRNAs and lncRNAs, provide an additional level of complexity to the epigenome of the ear. MiRNAs are a major class of regulatory RNA [44] and 60% of the human protein-coding genes are putative targets for miRNA, with many having CpG islands, which in turn can be epigenetically regulated [45]. In particular, mutations in miR-96 have now been established to be causally associated with progressive hearing loss in both humans and mice [45], resulting in severe transcriptional misregulation of hair cells, including defective expression of sound perception-related genes MYO15A and PTPRQ [32]. At the transcriptome-wide scale, more than 3200 lncRNAs have been discovered expressed in the mouse inner ear sensory epithelium, several of which display spatial and developmental specificity—indicative of their potential involvement in hearing-associated regulatory networks [10]. In age-related hearing loss, up-regulated lncRNAs—such as NONMMUT010961. 2—have been found to regulate oxidative stress response in cochlear cell models, supporting the involvement of ncRNAs in pathogenesis [1]. A recent review also emphasized the extensive implication of non-coding RNAs (miRNAs, lncRNAs and circRNAs) in hearing disability mechanisms, highlighting their rising potential as therapeutic targets [36,46].

However, translating non-coding RNA findings from model organisms to human diagnostics faces substantial challenges. Species differences in miRNA targeting, tissue-specific expression patterns, and the influence of genetic background complicate direct extrapolation [47]. Moreover, circulating miRNA profiles show high variability due to sample processing, normalization methods, and physiological fluctuations unrelated to hearing status. Longitudinal validation studies with standardized protocols across diverse populations are essential before ncRNA biomarkers can achieve clinical utility.

### 6.3. Epigenetic Modulation by Ageing, Noise Exposure, or Ototoxic Agents

Life course and environmental stressors play a major role in shaping the epigenetic landscape of hearing. Ageing changes the epigenome by altering somatic tissue DNA methylation and chromatin structure in a locus-specific or global manner: measures based on clocks of accelerated epigenetic ageing—such as GrimAge and PhenoAge—have been associated with hearing loss in older adults, supporting a connection between ear function and age-related changes to the epigenome [41]. Noise exposure is a well-known leading cause of acquired hearing loss, and newly associated heightened risk factors from epigenetic modifications are portrayed. Noise exposure induces coordinated epigenetic alterations, including DNA methylation and miRNA expression changes, which may serve as early molecular markers of cochlear stress [39,48,49]. In preclinical models, the DNMT1 inhibitor RG108 has shown otoprotective effects by reducing DNA damage and apoptosis in cochlear hair cells after noise exposure [50,51]; however, its translational relevance in humans remains to be established.

Additional chromatin-remodelling factors have also been implicated in cochlear stress responses [52].

### 6.4. Tissue- and Time-Specific Epigenetic Signatures: Diagnostic Potential and Challenges

Epigenetic marks display extreme tissue and temporal specificity to a degree that challenges underlying assumptions about peripheral biomarker validation. Methylation profiles in blood are influenced by leukocyte composition, immune activation, and systemic metabolic status—factors that might have nothing to do with cochlear biology [13,53]. Although periphery signatures and auditory phenotype may be related, no causality could be established. Suggested hypotheses are: (a) that there is a common developmental programming of haematopoietic and otic lines, (b) that systemic inflammation affects the two compartments, or (c) that there is statistical noise due to multiple comparisons. Confirmation would require a sample with paired inner ear and blood tissue; such testing is not feasible due to ethical and practical considerations in most settings. In the cochlea, these patterns replicate the development of hair cells and supporting cells from early differentiation to maturity. Peripheral methylation signatures show promise but require validation before they can be considered reliable proxies of inner ear biology [13,53]. Because direct longitudinal sampling of the living human cochlea is not feasible, animal models remain indispensable for defining the temporal and cell-type-specific epigenetic programmes that underlie auditory development, injury, and repair. Murine and other experimental systems allow paired analysis of cochlear tissue, peripheral blood, and functional hearing outcomes, enabling investigators to test whether epigenetic changes are causal, compensatory, or merely associative. They also provide a necessary platform for evaluating interventions such as DNA methylation inhibitors, histone-modifying agents, and regenerative reprogramming strategies before human translation. However, species-specific differences in cochlear regenerative capacity, developmental timing, and non-coding RNA regulation mean that animal findings should be interpreted as mechanistic guides rather than direct clinical surrogates.

About age-related impaired hearing, longitudinal studies point out that epigenetic age acceleration is associated with poorer auditory function, though the problem of cause and effect still awaits long-term follow-up in these patients. In addition, the issue of reproducibility and the impact of different patterns of expression between cell types and time points are questions that remain to be addressed when using non-coding RNA profiles as diagnostic aids. While tissue-level data from animal-derived models—miR-96 mutation and lncRNA expression dynamics—have provided a significant amount of information, for these findings to be translated into clinical assays in humans, validation in accessible (to tissue) matrices across different populations will be required [47].

Accordingly, peripheral blood methylation signatures should currently be considered hypothesis-generating biomarkers rather than validated clinical surrogates of cochlear pathology. Their future utility will depend on longitudinal replication, standardized preprocessing, adjustment for blood-cell composition, multi-ancestry validation, and, where feasible, triangulation with experimental models or tissue-specific molecular data.

## 7. Molecular Pathways Linking Genomic and Epigenomic Alterations Hearing Loss

### 7.1. Hair Cell Biology and Regeneration

Auditory hair cells are the final sensory effectors of the auditory pathway, and also the core biological target for hearing loss caused by genetic and epigenetic abnormalities. Genes that regulate the function of these cells interact with the regulatory programmes that determine the outcome of cochlear damage.

Auditory hair cells are the central mechanoreceptive elements of the inner ear that transduce vibrational stimuli into neural signals via the organ of Corti. Their developmental regulation is of critical importance: major signalling pathways have been identified (Notch, Hedgehog and Tbx2), driving the differentiation and fate commitment of hair cell subtypes, and manipulation of such pathways has allowed for targeted de novo regeneration for preclinical models.

Importantly, heteroplasmy of mitochondria—the presence within cells of both mutant and wild-type mtDNAs—also complicates the genotype–phenotype correlation. The m.1555A>G mutation in MT-RNR1, for instance, demonstrates a threshold effect whereby >60% heteroplasmy typically results in spontaneous hearing loss and <60% causes susceptibility without baseline impairment to aminoglycosides [30]. This non-linear relationship undermines simplistic genetic counselling and shows the requirements to implement heteroplasmy quantitative analysis in clinical diagnostics. Stem-cell-based approaches, including genetic delivery of cell-cycle regulators such as p27Kip1 through AAV vectors and pharmacological modulation, have shown potential to promote hair cell regeneration in preclinical models [41].

### 7.2. Mitochondrial Dysfunction and Oxidative Stress

This paper proposes that mitochondrial dysfunction and oxidative stress are the core mechanism linking genetic, environmental, and age-related factors to cochlear degeneration. The cochlea has high metabolic demand and limited regenerative capacity, so these related disruptions ultimately cause sensory cell damage and hearing decline.

Maintenance of mitochondrial health is essential for cochlear function because hair cells and spiral ganglion neurons are dependent on ATP consumption for various aspects of transduction and synaptic release. Mitochondrial dysfunction in the form of increased ROS and mtDNA damage is a central event that mediates age-related and noise-induced hearing loss [54]. The production of ROS through oxidative phosphorylation results in lipid peroxidation, DNA damage, and apoptotic cascades in cochlear cells. Of note, the mitochondrial unfolded protein response and mediator proteins such as OPA1—mediating cristae structure and fusion—maintain mitochondrial plasticity; involvement of OPA1 perturbs mitochondrial dynamics and increases hair cell susceptibility [55,56]. Moreover, the mitochondrial permeability transition pore (mPTP) plays an important role in apoptotic signalling. Abnormal opening results in ATP depletion and cytochrome c release, which initiates cell death. The newly discovered regulator ATAD3A reveals new targets for mPTP inhibition to alleviate hair cell injury [37].

### 7.3. Ion Channel, Synaptic, and Inflammatory Pathways

Previous studies often regarded the loss of sensory cells as the only core cause of hearing impairment. This paper points out that abnormalities in three types of pathways, including cochlear ionic homeostasis dysregulation, can also cause the disease. The interaction between genetic variations and epigenetic changes affects the timing of disease onset, the rate of disease progression, and the body’s response to damage.

Several molecular pathways identified through genomic studies converge on ion channel regulation and synaptic maintenance. For example, variants affecting SLC26A5 and cochlear amplification have been linked to outer hair cell electromotility via prestin-dependent mechanisms [57], while alterations in hair-bundle and stereociliary proteins contribute to disrupted mechanotransduction and cochlear integrity [58]. Potassium channel dysfunction and ribbon-synapse integrity influence cochlear amplification and neural transmission [59]. In parallel, age-related inflammatory and mitochondrial changes may further impair these systems and contribute to cochlear vulnerability [60]. These pathways are relevant because they represent downstream biological nodes through which genetic susceptibility, epigenetic regulation, and environmental injury may intersect. Recent evidence emphasizes the role of immune and inflammatory pathways in sensorineural hearing loss. TNF-α, IL-1β and other cytokine cascades are initiated upon cochlear damage, where the activation of macrophages induces inflammation leading to hair cell degeneration [8]. Especially, the NLRP3 inflammasome is activated by ROS and impairment of mitochondria, in turn inducing pyroptosis and exaggerating hearing loss. This process is further exacerbated by ageing to promote cochlear inflammation—termed inflammaging—where aberrant immune signalling, ion channel malfunction and loss of mitochondrial function interface [58,60]. Epigenetic changes coordinately regulate cochlear expression of inflammatory genes; chromatin acetylation and DNA methylation modify cytokine responsiveness and potentially serve as bridging mechanisms between immune activation and sensory cell susceptibility [61].

## 8. Diagnostic and Prognostic Innovations

### 8.1. Genomic Tools for Early Detection and Risk Profiling

Targeted next-generation sequencing (NGS) panels have significantly increased diagnostic sensitivity in pediatric hearing loss by identifying cases missed by conventional auditory evoked responses. Collaborative efforts such as the ClinGen Hearing Loss Expert Panel have further improved variant interpretation and clinical classification [62]. In addition, transcriptome analyses across different populations have identified dysregulated genes, including ICAM1, SLC1A1, and CD24, which may be associated with neurogenic hearing loss and represent potential emerging biomarkers [55].

Universal newborn hearing screening relies primarily on otoacoustic emissions and automated auditory brainstem responses, which remain essential for early identification but do not establish the underlying etiology. In this context, genomic testing should be considered complementary: when integrated after failed screening or in high-risk infants, targeted NGS may improve etiological diagnosis, refine prognosis, and support personalized counselling. Although integration of genetic testing into screening pathways increases diagnostic yield, its implementation requires careful consideration of cost, variant interpretation, and ethical implications [63].

### 8.2. Epigenetic Biomarkers: Current Status and Future Scope

The search for epigenetic biomarkers in hearing loss is gaining traction, but clinical translation remains premature. Epigenome-wide studies have identified methylation changes associated with nonsyndromic and age-related hearing loss, including blood-based signatures linked to auditory phenotypes. However, these findings are challenged by tissue specificity, residual confounding, differences in blood-cell composition, and the difficulty of inferring cochlear mechanisms from peripheral samples. Although recent large-scale studies have reported candidate CpG sites associated with age-related hearing impairment, these observations should currently be interpreted as promising discovery signals rather than clinically actionable biomarkers [13,51,64].

### 8.3. Multi-Omic Integration and Bioinformatic Frameworks: Current Status and Future Scope

The integration of multi-omic layers—genomics, epigenomics, transcriptomics, and, increasingly, proteomics—is reshaping diagnostic paradigms in hearing loss. Genomic testing can identify inherited susceptibility or causal variants, whereas epigenomic profiling may capture environmentally responsive or disease-state-dependent molecular signatures that are not evident from DNA sequence alone. Their combined interpretation may therefore improve risk stratification, variant prioritization, biomarker discovery, and therapeutic selection, particularly in multifactorial or age-related forms of hearing loss. Platforms such as the gEAR portal facilitate centralized visualization and analysis of inner ear single-cell and multi-omic datasets, accelerating discovery and hypothesis generation across the research community [62]. Machine learning pipelines capable of integrating variant calls, methylation signatures, and expression patterns represent a promising route toward precision audiology, although clinical translation will require external validation, ancestry-diverse datasets, and standardized analytical workflows.

### 8.4. Ethical, Legal and Psychosocial Implications of Genomic Testing in Hearing Loss

#### 8.4.1. Genetic Counselling and Recurrence Risk

Genetic counselling represents a central component of genomic testing in hearing loss, as new diagnoses require careful communication of recurrence risks and inheritance patterns. Recurrence risk is approximately 25% in autosomal recessive hearing loss, may reach up to 50% in autosomal dominant forms depending on penetrance, and follows maternal inheritance in mitochondrial disorders.

Counselling must also address variable expressivity and incomplete penetrance, which complicate phenotype prediction in several autosomal dominant genes such as *MYO15A*, *COCH*, and *TECTA*. In contrast, genes such as *GJB2* and *OTOF* are often associated with high or near-complete penetrance, enabling more accurate prognostic counselling. These genetic characteristics directly influence risk assessment and family planning discussions.

#### 8.4.2. Psychosocial Implications and Reproductive Decision-Making

Genomic and genetic testing in hearing loss is frequently associated with significant psychosocial implications, including concerns regarding autonomy, privacy, potential discrimination, and the emotional impact of genetic results on patients and families [65].

Genetic counselling should therefore include education regarding reproductive options, such as carrier testing of partners, preimplantation genetic diagnosis (PGD), and targeted prenatal testing in families with known pathogenic variants. These interventions may facilitate informed reproductive decision-making, but they may also introduce ethical complexity and emotional burden, particularly in cases with variable or uncertain prognosis.

#### 8.4.3. Ethical, Legal and Societal Considerations

Broader ethical and legal frameworks play a critical role in governing the use of genomic information. Policies such as the U.S. Genetic Information Non-Discrimination Act (GINA) and the European Union General Data Protection Regulation (GDPR) aim to protect individuals from misuse of genetic data; however, their scope and enforcement are not uniform across jurisdictions and may remain incomplete in relation to employment and healthcare contexts [66].

In addition, the field of genetic counselling faces structural challenges, including workforce shortages that are particularly pronounced in low- and middle-income countries, where access to audiogenetic services remains limited [67,68]. Beyond clinical practice, ethical debates also address the societal implications of large-scale genomic testing, including concerns about stigmatization, cultural attitudes toward genetic conditions such as deafness, and tensions between disability rights frameworks and preventive genomic medicine. These issues are framed within international human rights perspectives, including the United Nations Convention on the Rights of Persons with Disabilities [69].

New diagnoses require a consideration of ethical, legal and psychosocial issues.

Genetic counselling should clearly indicate the possibility of recurrence: 25% for autosomal recessive HL, up to 50% for autosomal dominant HL depending on penetrance, and maternal inheritance for mitochondrial mutations. Genomic and genetic testing is often associated with fears of autonomy, privacy, discrimination and psychological implications of results [65]. Families must be educated about reproductive options, such as carrier testing of partners, preimplantation genetic diagnosis (PGD), and targeted prenatal testing in families with known pathogenic variants. Counselling must further address variable expressivity and penetrance for genes such as MYO15A, COCH and TECTA that render phenotype prediction challenging. The degree of penetrance differs widely for hearing loss genes. Many autosomal dominant hearing loss genes, such as TECTA, COCH and WFS1, are associated with either low or age-related penetrance of disease that renders the prediction of risk to family members difficult. On the contrary, recessive genes such as GJB2 and OTOF have nearly complete penetrance, which is useful in providing more accurate prognosis counselling.

Global policies such as the U.S. Genetic Information Non-Discrimination Act (GINA) and EU General Data Protection Regulation (GDPR) are seeking to protect individuals from improper use of their genetic information, though these protections are not uniform and may be incomplete with respect to health care and employment [66]. The profession of genetic counselling also needs to evolve to meet the demands associated with more complex genomic information; however, workforce shortages remain and are particularly pronounced in low- and middle-income countries where audiogenetic resources are scarce [67]. Counselling faces challenges of reproductive control, stigmatization and cultural attitudes to genetic conditions such as deafness. Scoping reviews highlight concerns that population-based genomics may undermine any value of disability through the celebration of attitudes to prevent buttressing ideals in relation to diversity and inclusion now embedded within human rights frameworks, such as the UN Convention on the Rights of Persons with Disabilities [69].

## 9. Therapeutic and Translational Advances

### 9.1. Gene Therapy and Precision Genome Editing (e.g., CRISPR, Base Editing)

**Figure 2:**
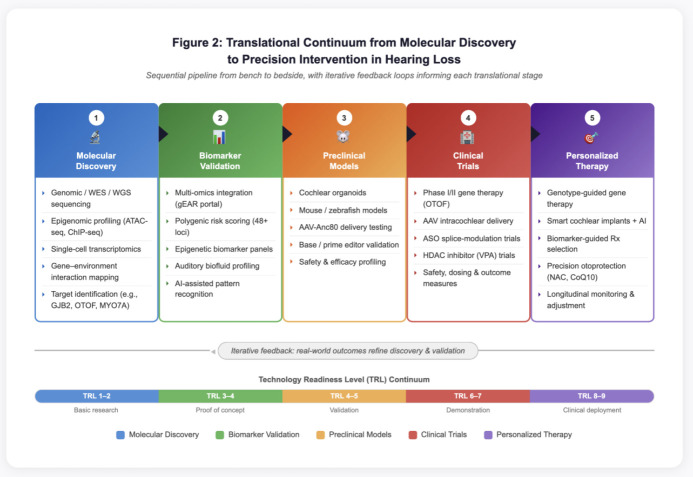
Translational continuum from molecular discovery to precision intervention in hearing loss. Preclinical models continue to set the stage for potential treatment: CRISPR-based base editing of the OTOF gene in mice showed restored expression (~88% of inner hair cells) of otoferlin, resulting in sustainable auditory responses with low off-target effects at well over 18 months [70,71,72]. More comprehensive reviews highlight the broadening armamentarium—that is, CRISPR-Cas9 nucleases, base editors, and prime editors—for correction of various monogenic deafness causing mutations with increasing focus on cochlear safety and delivery device [73,74]. Nanotechnology-based gene delivery systems are also being developed, which provide better targeting to the cochlear sensory cells and may overcome physical barriers for inner ear delivery [75].

For hereditary hearing loss, gene therapy is rapidly moving from “experiment” to “clinic.” Notable clinical advancements have been made especially in the case of OTOF-associated deafness (DFNB9): for the treatment of otoferlin-related severe progressive neuron disorder, AAV-mediated delivery of genes resulted in a robust recovery in auditory function among children—ten out of eleven patients treated have recovered hearing profiles and some even reached near-normal thresholds and spoken word recognition [69] (Figure 2).

### 9.2. Epigenetic Modulators (HDAC Inhibitors, DNA Demethylating Agents)

Modulation of epigenetic pathways represents a promising experimental strategy that may eventually complement genetic therapies, although current evidence remains largely preclinical. Histone deacetylase inhibitors such as valproic acid (VPA) are also identified as inner ear gene expression modulators; preclinical models have shown potential for treating genetic hearing loss, associated with KCNQ4 mutations, when used in combination with agents like CHIR99021 [76]. Indeed, HDAC dysregulations have been implicated in different forms of deafness (e.g., noise-induced, ototoxic, and age-related SNHL), which has placed epigenetic inhibitors on the longlist of potential targets for extended otoprotection strategies [77]. Therapeutic potential, along with small molecule accessibility, also raises the idea of repurposing existing compounds in auditory medicine, potentially providing systemic or localized manipulation of chromatin states for inner ear function maintenance.

### 9.3. RNA-Based Platforms (ASOs, SiRNAs, mRNA Therapeutics)

RNA-based approaches represent an emerging therapeutic strategy for genetic hearing loss. New approaches involve antisense oligonucleotides (ASOs) and mRNA-based therapies to restore splicing aberrations or to overwrite them with deleterious mutations in cochlear genes. While the hearing loss literature is relatively young, the advances in oligonucleotide chemistry that would facilitate cell uptake, stability and targeting are already here, pointing to a straightforward translational route [76]. In addition, CRISPR-based RNA editing systems are being explored to convert pathogenic RNAs without modifying genomic DNA reversibly, perhaps more safely in sensitive tissues such as the inner ear [74]. These breakthroughs indicate a future in which transient RNA therapeutics may offer dynamic and repeatable opportunities for treating hearing loss diseases.

### 9.4. Drug Testing Using Stem-Cell Models and Organoids

Stem-cell-based models, e.g., cochlear organoids and inner ear sensory epithelium cultures, are increasing in importance due to their translational relevance. These systems provide human-relevant test beds for assessing gene therapies, epigenetic drugs and RNA-based agents to facilitate high-content screens for effectiveness and safety [78]. Together with CRISPR-engineered models, organoid systems offer direct modelling of genetic hearing loss and enable high-throughput screening of candidate interventions. While these tools are still early in application, they hold the promise to decrease animal model use, expedite target validation and facilitate precision audiology development pipelines.

### 9.5. Individualized Audiology and Comprehensive Management Options

The future of hearing loss management is likely to involve individualized care pathways in which molecular diagnosis, biologic therapies, assistive technologies, and genetic counselling are combined according to disease mechanism and patient need [79,80]. Device-based innovations, including intelligent hearing aids, may serve as useful adjuncts to biologically targeted interventions, particularly for patients with multifactorial or irreversible forms of hearing loss [81,82,83,84,85]. However, these technologies should be regarded as complementary clinical tools rather than central components of the genomic and epigenomic framework discussed in this review.

## 10. Conclusions

This review highlights hearing loss as a growing global health challenge and emphasizes how advances in genomics and epigenomics are reshaping its biological understanding, diagnosis, and treatment. Genetic studies have refined the architecture of monogenic and polygenic disease, whereas epigenetic research has revealed regulatory mechanisms that may link development, ageing, environmental exposure, and therapeutic response. Emerging approaches—including gene therapy, RNA-based platforms, organoid models, and integrated multi-omics—offer substantial translational promise, but their clinical impact will depend on rigorous validation, equitable implementation, and inclusion of diverse populations. Aligning molecular innovation with public health priorities will be essential to reduce the burden of disabling hearing loss worldwide.

## Figures and Tables

**Table 1:** High-yield monogenic deafness genes and associated inheritance patterns. Original author-generated synthesis based on the cited literature.

Gene	Protein/Function	Associated Hearing Loss Phenotype	Inheritance Pattern	Diagnostic Yield Contribution
GJB2	Connexin-26 gap junction	AR nonsyndromic severe–profound SNHL	Autosomal recessive	Highest worldwide (up to 50% in some cohorts)
STRC	Stereocilin	AR mild–moderate SNHL	Autosomal recessive	High yield (5–15% depending on population); CNV-driven
OTOF	Otoferlin	Auditory neuropathy spectrum disorder (ANSD)	Autosomal recessive	Major cause of ANSD; 2nd most common AR HL gene
SLC26A4	Pendrin (anion transporter)	Pendred syndrome; EVA-associated HL	Autosomal recessive	Leading cause of EVA and syndromic mimics
MYO15A	Myosin-XVa	Severe–profound congenital SNHL	Autosomal recessive	Important contributor in consanguineous families
TECTA	α-Tectorin	Mid-frequency (“cookie-bite”) HL	Autosomal dominant or recessive	Frequent AD cause of stable mid-frequency HL
COL11A2	Collagen XI chain	Nonsyndromic SNHL; part of tectorial membrane	Autosomal dominant	Contributes to progressive AD HL
TMC1	Transmembrane channel-like 1	Severe–profound congenital HL	AD or AR	High prevalence in several founder populations
USH2A	Usherin	Usher syndrome type 2	Autosomal recessive	Major gene for deaf-blindness; often appears in HL panels
MT-RNR1	Mitochondrial rRNA	Aminoglycoside-induced HL	Maternal (mitochondrial)	Clinical impact for drug contraindications

**Table 2:** Major epigenetic mechanisms and their roles in auditory biology. Original author-generated synthesis based on the cited literature.

Epigenetic Mechanism	Description	Auditory System Roles	Examples/Evidence
DNA Methylation	Addition of methyl groups to CpG sites, repressing transcription	Regulates cochlear development, stress responses, ageing-related decline	Differential methylation found in NIHL, ARHL, mitochondrial pathways
Histone Modifications	Acetylation, methylation, phosphorylation altering chromatin accessibility	Controls hair cell differentiation and regeneration potential	HDAC activity implicated in ototoxicity; H3K9 methylation affects hair cell survival
Chromatin Remodelling Complexes	ATP-dependent modification of chromatin structure	Enables transcription of hair cell identity genes	SWI/SNF components essential for cochlear morphogenesis
MicroRNAs (miRNAs)	Post-transcriptional repression of mRNA	Fine-tune hair cell gene expression, mechanotransduction, synaptogenesis	miR-96 mutations cause DFNA50; noise alters miR-183 family
Long Non-Coding RNAs (lncRNAs)	Regulate transcriptional programmes and chromatin state	Involved in otic lineage specification and cellular stress pathways	lncRNA Gas5 and others implicated in cochlear oxidative-stress responses
RNA Editing	A-to-I editing alters transcript sequence/function	Modulates synaptic transmission and ion channel physiology	ADAR-dependent editing changes shown in auditory brainstem pathways
3D Genome Architecture	Looping and higher-order genome structure shaping gene expression	Regulates enhancer–promoter interactions in developing cochlea	Structural epigenomics studies show altered loops in HL models

## Data Availability

This review article does not report any new original data. All data discussed are derived from previously published studies, which are cited throughout the manuscript and publicly available from the original sources.

## Abbreviations

The following abbreviations are used in this manuscript:

HL	Hearing loss
ASPR	Age-standardized prevalence rate
SDI	Socio-demographic index
YLDs	Years lived with disability
LMICs	Low- and Middle-Income Countries
NIHL	Noise-induced hearing loss
NSHL	Nonsyndromic hearing loss
PRS	Polygenic risk scores
GINA	Genetic Information Non-Discrimination Act
GDPR	General Data Protection Regulation
VPA	Valproic acid
gEAR	Genomics-Expression Analysis Resource
AR	Autosomal recessive
AD	Autosomal dominant
SNHL	Sensorineural hearing loss
CNV	Copy-number variant
WES	Whole-exome sequencing
WGS	Whole-genome sequencing
NGS	Next-generation sequencing
VUS	Variant of uncertain significance
EVA	Enlarged vestibular aqueduct
ANSD	Auditory neuropathy spectrum disorder
PRS	Polygenic risk score
AAV	Adeno-associated virus
HDAC	Histone deacetylase
DNMT	DNA methyltransferase
ASO	Antisense oligonucleotide
lncRNA	Long non-coding RNA
miRNA	microRNA
